# Protective Effect of Tetrandrine on Sodium Taurocholate-Induced Severe Acute Pancreatitis

**DOI:** 10.1155/2015/129103

**Published:** 2015-10-08

**Authors:** Xian-lin Wu, Jie-xing Li, Zhen-dong Li, Da-sheng Liu, Su-hong Lu, Kang-li Liu, Hong-yan Duan, Yu-hong Luo

**Affiliations:** ^1^Pancreatic Disease Center, The First Affiliated Hospital of Jinan University, Guangzhou 510632, China; ^2^Clinical Medicine Research Institute, The First Affiliated Hospital of Jinan University, Guangzhou 510632, China; ^3^Department of Traditional Chinese Medicine, Medical College of Jinan University, Guangzhou 510632, China; ^4^Department of Hepatobiliary Surgery, The First Affiliated Hospital of Jinan University, Guangzhou 510632, China; ^5^Department of Internal Medicine, Nancheng Peoples' Hospital, Dongguan 523071, China; ^6^Department of Fetal Medicine, The First Affiliated Hospital of Jinan University, Guangzhou 510632, China

## Abstract

Tet is a type of alkaloid extracted from *Stephania tetrandra*, and it has recently been demonstrated that Tet can protect against inflammation and free radical injury and inhibit the release of inflammatory mediators. The present study was designed to observe the protective effect of Tet on sodium taurocholate-induced severe acute pancreatitis (SAP). The rat model of SAP was induced by retrograde bile duct injection of sodium taurocholate and then treated with Verapamil and Tet. The results showed that Tet can reduce NF-*κ*B activation in pancreas issue, inhibit the SAP cascade, and improve SAP through inducing pancreas acinar cell apoptosis and stabilizing intracellular calcium in the pancreas, thus mitigating the damage to the pancreas. Our study revealed that Tet may reduce systemic inflammatory response syndrome (SIRS) and multiple organ dysfunction syndromes (MODS) to protect against damage, and these roles may be mediated through the NF-*κ*B pathway to improve the proinflammatory/anti-inflammatory imbalance.

## 1. Introduction

Acute pancreatitis (AP) is an acute abdominal condition. There are two types of AP, mild and severe. Mild AP, which accounts for 80% of the cases of AP, is self-limiting. However, approximately 20% of the cases of AP are severe [1]. Severe acute pancreatitis (SAP) is a life-threatening condition with a high mortality rate that progresses rapidly and is associated with many complications. The pathological features of SAP include extensive pancreatic hemorrhage and large areas of necrotic tissue [2]. Clinically, SAP is often accompanied by systemic inflammatory response syndrome (SIRS), acute respiratory distress syndrome (ARDS), acute lung injury (ALI), acute renal insufficiency, and hepatic impairment. Multiple organ dysfunction syndromes (MODS) can develop in the later stages of SAP, and uncontrolled SAP can ultimately cause multiple organ failure (MOF) [3]. The mortality rate for SAP is as high as 20% to 30% [4].

Currently, there are no effective medications for treating SAP. The main therapeutic approaches for this disease are symptomatic treatments, which include gastric decompression, the provision of pain relief, and the correction of fluid, electrolyte, and pH balances [5]. Many researchers are screening active ingredients from traditional Chinese medicine to assess the potential of these ingredients to treat SAP [6–8]. Tetrandrine (Tet), a monomer extracted from Radix Stephaniae Tetrandrae, is a nonselective calcium channel antagonist [9]. The anti-inflammatory effects of Tet have gradually attracted increasing research attention [10]. Tet has particularly salutary effects with respect to preventing and treating acute and chronic inflammation, inhibiting the release of inflammatory mediators, regulating inflammatory cell function, and protecting against free radical damage [11].

Given the significant anti-inflammatory effects of Tet, we intend to utilize this drug for the treatment of SAP. In this study, a stable animal model of SAP and SAP-associated organ damage was established. Tet intervention was then utilized to observe the protective effects of Tet treatment against SAP and SAP-related organ damage as well as the possible mechanisms of these effects.

## 2. Materials and Methods

### 2.1. Drugs

**Figure 1:**
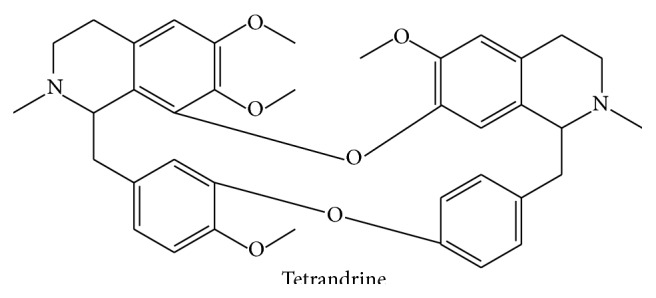
Chemical structure of Tetrandrine (C_38_H_42_N_2_O_6_).

Tetrandrine and standard substance (molecular formula: C_38_H_42_N_2_O_6_) were purchased from Institute of Chinese Materia Medica, Nanjing (Figure 1).

### 2.2. Animal Groups

A total of 120 male specific pathogen-free (SPF) Sprague-Dawley (SD) rats were randomly divided into five groups: the normal group (the N group), the sham-operation group (the S group), the SAP model group (the M group), the Tet treatment group (the Tet group), and the Verapamil (Ver) treatment group (the Ver group). Random allocation was used to divide each group into three subgroups (for the 3 h, 6 h, and 12 h time points), with *n* = 8 for each subgroup.

### 2.3. Animal Modeling and Drug Dosing

The experimental rats were anesthetized with a 3 mL/kg intraperitoneal injection of 10% chloral hydrate. After the abdominal cavity was exposed, the intestine along the mesenteric border of the duodenum was punctured with a 3.5-gauge flat needle. This needle was retrogradely inserted approximately 0.5 cm into the ampullary opening of the biliopancreatic duct and used to inject a 1 mL/kg dose of 5% sodium taurocholate into this duct at a rate of 0.2 mL/min. Following this injection, a small artery clamp was used to occlude the duct for 2 minutes; subsequently, the punctures in the duodenal wall were sutured, and the surgical incision was closed with a double layer of stitching. The pancreas and duodenum treatments were switched in the S group. After the operation, the rats in each group were subcutaneously injected with 30 mL/kg of 0.9% NaCl. The rats were provided with water ad libitum after surgery [12].

In accordance with the results of preliminary experiments, after the SAP model had been induced, intraperitoneal injections of 40 mg/kg Tet and 1 mg/kg Ver were administered to rats in the Tel and Ver groups, respectively, and rats in the M group received an intraperitoneal injection of an equal volume of 0.9% NaCl. Rats in each group received a postoperative 30 mL/kg subcutaneous injection of 0.9% NaCl and were provided with water ad libitum after surgery. The three observation time points of 3 h, 6 h, and 12 h were established.

### 2.4. Detecting Wet/Dry Weight Ratios and Pathological Scores for Pancreatic Tissue

The wet weight of the body of the pancreas of each rat was determined. The dry weight of each of these pancreas samples was determined by incubating the samples in a 60°C oven for 72 hours until constant weights were attained. These two types of weights were then used to calculate wet/dry weight ratios for pancreatic tissue. Pancreatic tissue samples were also obtained for paraffin sectioning and hematoxylin-eosin (HE) staining. Appropriate histological scoring methods were adopted to determine pathological scores, with higher pathological scores indicating more severe tissue damage.

### 2.5. Detecting Serum Amylase Activity and the Myeloperoxidase (MPO) Activity of Pancreatic Tissue Homogenates

Using an automatic biochemical analyzer (Hitachi 7600), *α*-amylase (AMY) levels in the peripheral blood were measured in accordance with the procedures specified by an AMY activity assay kit. A colorimetric assay was used to determine MPO activity in pancreatic tissue homogenates. To eliminate edema-related effects, the MPO activity per gram of dry tissue was adopted as a measure that could relatively accurately represent the extent of neutrophil infiltration. In particular, the following equation was utilized: MPO units/g dry tissue = MPO units/g wet tissue × wet/dry weight ratio of the tissue.

### 2.6. Detecting Apoptosis and Necrosis in Pancreatic Acinar Cells

Single-cell suspensions of pancreatic acinar cells were prepared. To detect apoptosis in these suspensions, fluorescein isothiocyanate- (FITC-) labeled annexin V was used as a probe for flow cytometry and fluorescence microscopy observations; this approach relies on the fact that annexin V specifically binds to phosphatidylserine (PS), which translocates to the outer leaflet of the cell membrane during apoptosis. The nucleic acid dye propidium iodide (PI) can pass through the cell and nuclear membranes of cells in the middle and late stages of apoptosis, marking these cells with red fluorescence. The combined use of annexin V and PI can therefore successfully differentiate between early apoptotic cells, late apoptotic cells, and necrotic cells.

### 2.7. Detecting Calcium Ion Levels within Pancreatic Acinar Cells and the Transporter Activation of Nuclear Factor-Kappa B (NF-*κ*B) in Pancreatic Tissue

Single-cell suspensions of pancreatic acinar cells were prepared, and the cell density of these suspensions was adjusted to 5 × 10^8^ cells/L. A 2 mL sample of pancreatic acinar cell suspension was obtained, Fluo-2/AM was added to a final concentration of 5 *μ*mol/L, and the resulting sample was placed in a 37°C water bath and incubated in the dark for 30 to 45 minutes. Flow cytometry was then used for the detection of fluorescence. To assess NF-*κ*B activity, pancreatic tissue was immersed in a 4% paraformaldehyde solution (at 4°C). Frozen sections were prepared after conventional embedding and fixing procedures were performed. NF-*κ*B was measured in accordance with the procedures provided in an NF-*κ*B activation assay kit. The following formula for the NF-*κ*B activation rate was utilized for statistical analysis: NF-*κ*B activation rate = (Total number of positive cells)/(Total number of cells) × 100%.

### 2.8. Statistics

All the data are expressed as the means ± SEM. The means of different groups (same times) were compared using a one-way analysis of variance (ANOVA). Differences were assumed to be significant at *P* < 0.05. The data were ranked using a nonparametric rank sum test, and the death rates of different groups were compared using fourfold table chi-square test.

## 3. Results

### 3.1. Pathological Changes in Pancreatic Tissue and the Pathological Scores of Each Examined Organ

**Figure 2:**
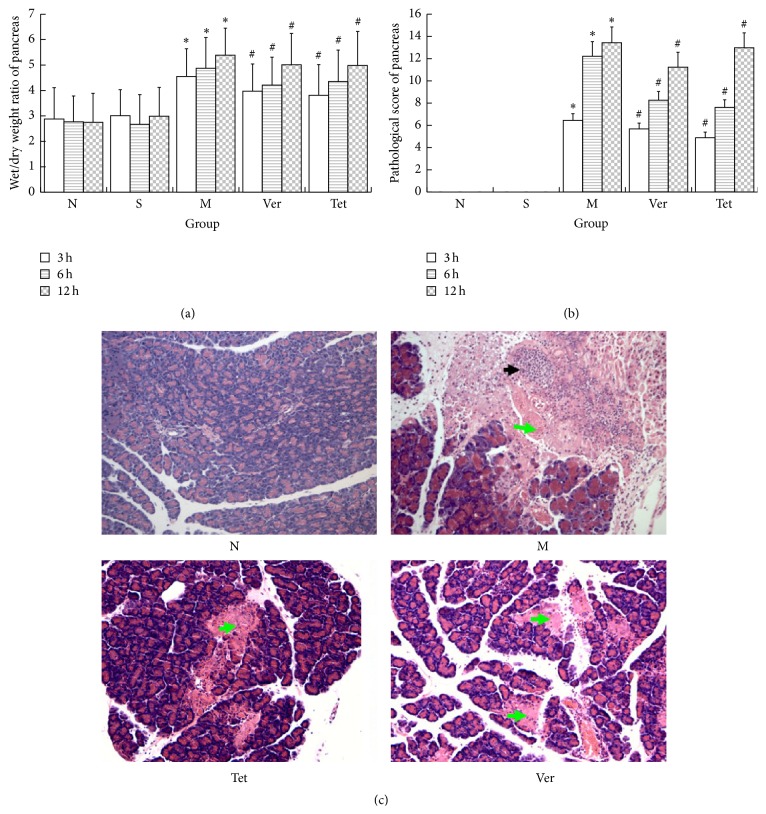
Pathological sections and scores of pancreatic tissues. (a) Chart comparing the wet/dry weight ratios of pancreatic tissue. The wet/dry ratios decreased significantly after therapeutic intervention; (b) pathological scores of pancreatic tissue samples. The pathological scores of the pancreas were significantly increased after model was established, whereas the pathological scores were significantly lower in Tet treatment group; (c) microscopic images of pathological sections of pancreatic tissue (100x). The structure of pancreatic cells was integrated in normal group, and the intercellular space was normal, and there were no signs of either hemorrhagic necrosis or the infiltration of inflammatory cells. In other groups, lobular spaces widened, acinar hemorrhage and edema were evident, large areas of necrosis were present, the parenchymal and interstitial infiltration of numerous inflammatory cells occurred, and fat necrosis lesions could be observed. However, the aforementioned phenomena were somewhat less severe in the Tet groups (^*∗*^ *P* < 0.05 versus normal group; ^#^ *P* < 0.05 versus model group).

In the S group, no significant changes in pancreatic tissue at any time point were observed. In the M group, edema, hemorrhage, inflammatory cell infiltration, and necrosis occurred in pancreatic tissue. These changes were accompanied by corresponding increases in the wet/dry weight ratios and pathological scores of the pancreatic tissue. Compared with the M group, the Ver and Tet groups exhibited reduced pathological changes and lower pathological scores (Figure 2).

### 3.2. Determinations of Serum Amylase and MPO Activity in Pancreatic Tissue Homogenates

**Figure 3:**
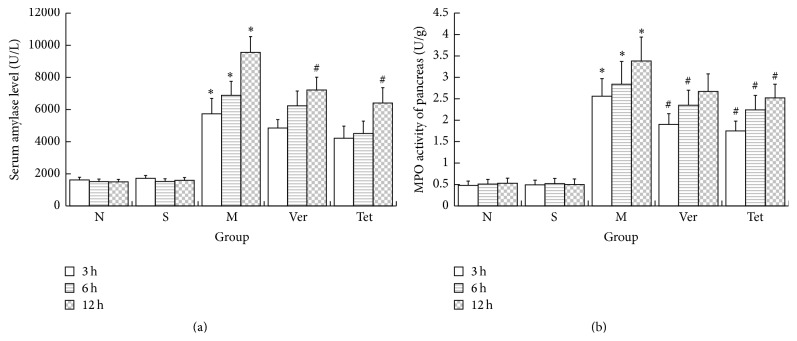
Serum amylase level and pancreatic MPO activity. (a) Serum amylase level. After the establishment of the SAP model, serum amylase was significantly elevated in the M group relative to the N and S groups but was lower in the Ver and Tet intervention groups than in the M group; (b) MPO activity of pancreatic tissue homogenates. After the establishment of the SAP model, the MPO activity of the pancreatic tissue homogenates was significantly elevated in the M group relative to the N and S groups but was lower in the Ver and Tet intervention groups than in the M group (^*∗*^ *P* < 0.05 versus normal group; ^#^ *P* < 0.05 versus model group).

In the S group, both serum amylase activity and MPO activity remained stable at each examined time point. Serum amylase activity was significantly higher in the M group than in the S group at each time point after the SAP model had been successfully induced. Serum amylase activity and MPO activity gradually increased over time. At each examined time point, serum amylase activity and MPO activity were lower in the Ver and Tet groups than in the M group. These results demonstrated that serum amylase activity and pancreatic MPO activity decreased after Tet or Ver treatments, suggesting that Tet and Ver can protect pancreatic tissue by alleviating pancreatic injury and reducing neutrophil infiltration (Figure 3).

### 3.3. The Detection of Pancreatic Acinar Cell Apoptosis and Necrosis

**Figure 4:**
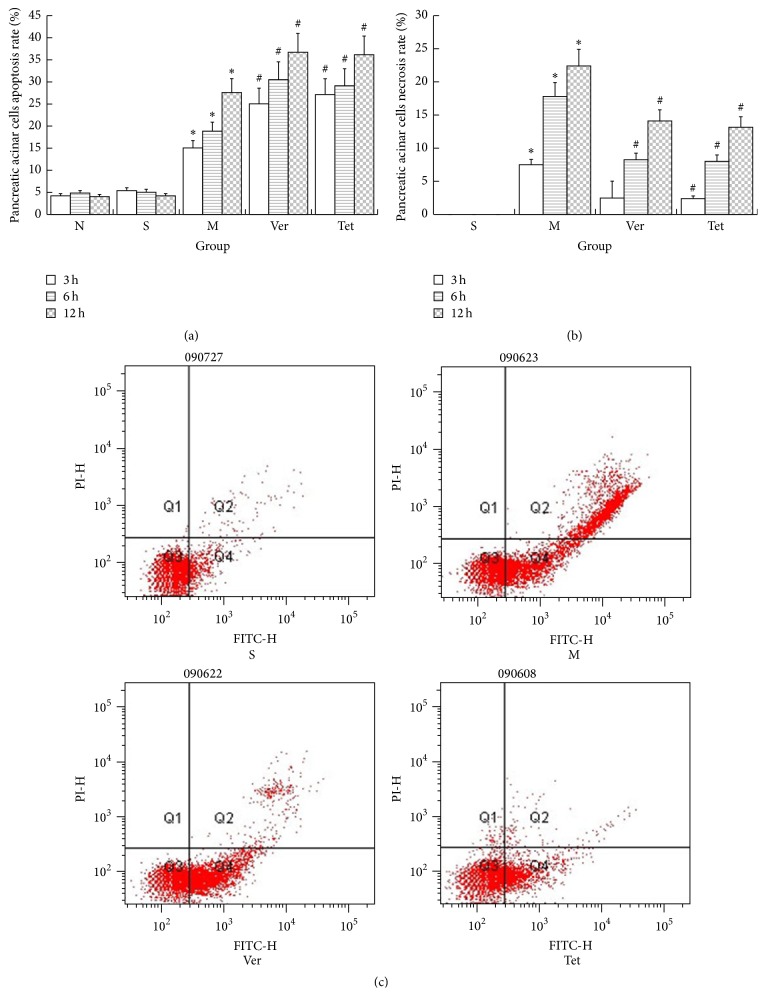
Apoptosis and necrosis of pancreatic acinar cells. (a) Apoptosis rates of pancreatic acinar cells. The apoptosis rates of pancreatic acinar cells were significantly higher in the M group than in the N and S groups. The groups treated with Ver or Tet exhibited higher apoptosis rates than the M group; (b) necrosis rates of pancreatic acinar cells. Necrosis of pancreatic cells was clearly evident after the induction of the SAP model, and necrosis rates were lower among cells treated with Ver or Tet than among untreated cells; (c) flow cytometry plots of acinar cell apoptosis (^*∗*^ *P* < 0.05 versus normal group; ^#^ *P* < 0.05 versus model group).

In the S group, apoptosis was observed to a certain extent among pancreatic acinar cells at each examined time point; however, the apoptosis rate was not high, and no significant necrosis was observed. In the M, Ver, and Tet groups, both apoptotic and necrotic pancreatic acinar cells were present at each time point. Moreover, at each time point, there were significantly higher numbers of apoptotic and necrotic cells in each of these three groups than in the S group. The number of apoptotic and necrotic cells increased over time in the M, Ver, and Tet groups. Relative to the M group, the Ver and Tet groups had significantly more apoptotic cells but significantly fewer necrotic cells at each time point. These findings suggested that, among pancreatic acinar cells in cases of SAP, the Tet and Ver treatments significantly reduced necrosis, resulting in an increased number of apoptotic cells and surviving cells. Thus, in the context of SAP, Tet and Ver can alleviate pancreatic necrosis and promote cellular apoptosis (Figure 4).

### 3.4. The Detection of Calcium Ion Levels in Pancreatic Acinar Cells

The fluorescence intensities of calcium ions within pancreatic acinar cells were greater in the Ver and Tet groups than in the S group at each time point, and these fluorescence intensities increased over time. The fluorescence intensities of calcium ions were lower in the Ver and Tet groups than in the M group at each time point. The differences in these intensities between the Ver group and the Tet group at each time point were not significant. These results demonstrated that, in the context of SAP, the fluorescence intensity of calcium ions within acinar and other pancreatic cells and pancreatic acinar cells was significantly reduced after receiving Tet or Ver treatment, suggesting that Tet and Ver could stabilize the intracellular calcium ion levels of acinar cells, reducing calcium overload and mitigating the damage to pancreatic cells.

### 3.5. The Detection of NF-*κ*B Transporter Activation in Pancreatic Tissue

**Figure 5:**
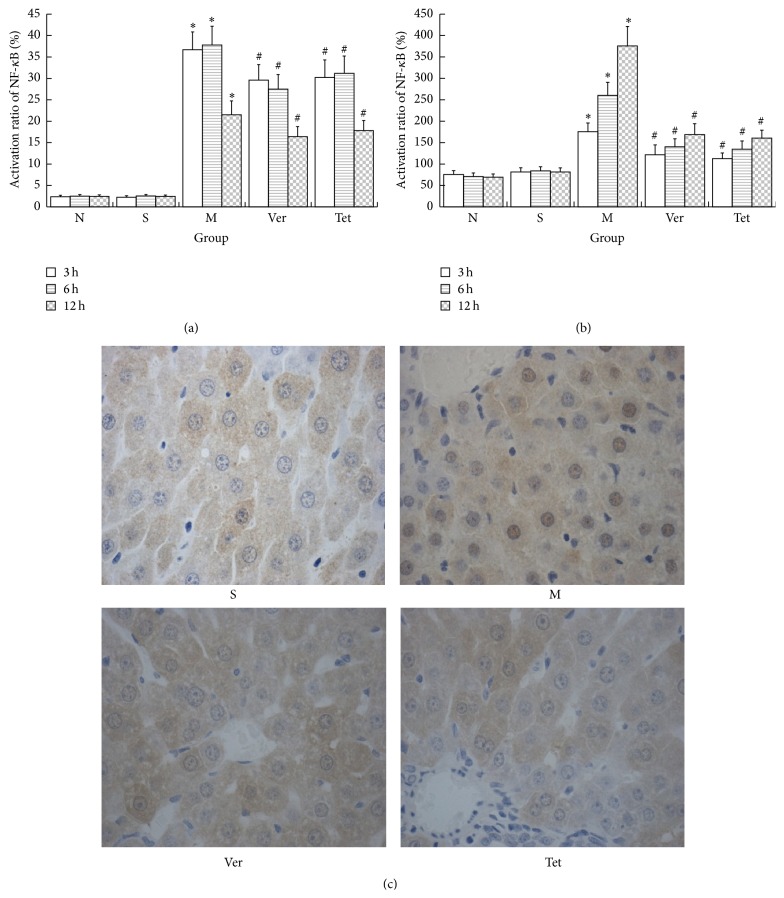
Calcium ion levels and NF-*κ*B transporter activation rates in pancreatic tissue and liver. (a) NF-*κ*B translocation rates in pancreatic tissue. TNF-*κ*B transporter activation rates in pancreatic tissue were lower in SAP rats treated with Ver or Tet than in untreated SAP rats; (b) calcium ion levels in liver. Calcium ion levels were lower in SAP rats treated with Ver or Tet than in untreated SAP rats. (c) Histological images indicating NF-*κ*B transporter activation in pancreatic tissue (^*∗*^ *P* < 0.05 versus normal group; ^#^ *P* < 0.05 versus model group).

In the S group, NF-*κ*B transporter activation was maintained at stable, low levels throughout the experiment, and activation rates were less than 3%. Within this group, the activation rate at each time point was not significantly different. NF-*κ*B transporter activation rates were significantly higher in the M, Ver, and Tet groups than in the S group at each time point. This rate reached a peak during the 3–6 h time period and subsequently decreased. NF-*κ*B transporter activation rates were lower in the Ver and Tet groups than in the M group, suggesting that Tet or Ver treatment for SAP significantly decreased these rates in pancreatic tissue. This finding indicated that Tet and Ver can inhibit NF-*κ*B transporter activation in pancreatic tissue in the context of SAP (Figure 5).

## 4. Discussion

Tet, which is also known as Tetrandrine hormone, has a wide range of pharmacological effects. (1) Ca^2+^ antagonism: Tet can act as a calcium antagonist by inhibiting extracellular Ca^2+^ influx, regulating the distribution of intracellular Ca^2+^, and maintaining intracellular Ca^2+^ homeostasis [13]. (2) Immunosuppressive effects: Tet can inhibit I*κ*B (inhibitor of kappa B) degradation, inhibiting NF-*κ*B transporter activation and the expression of NF-*κ*B-dependent genes. It can thereby significantly reduce the generation of the proinflammatory cytokines tumor necrosis factor-alpha (TNF-*α*) and interleukin-6 (IL-6). Moreover, Tet inhibits the synthesis of antibodies by mitogen-stimulated B cells, promotes lymphocyte proliferation, and influences natural killer (NK) cell-mediated cytotoxicity [14]. (3) Tet acts as a free radical scavenger. Tet also inhibits the production of platelet activating factor, interleukin-1 (IL-1), prostaglandin (PG), and other inflammatory cytokines, thereby hampering the orientation, chemotaxis, and phagocytic activity of inflammatory cells and producing significant anti-inflammatory effects [15]. Therefore, in theory, Tet can interfere with many different aspects of the pathological process of AP; for example, Tet could affect initiating factors of AP, produce anti-inflammatory responses, and improve microcirculation, among other possibilities. Few studies have examined the use of Tet for the treatment of SAP. However, research has determined that, in rats, Tet can inhibit inducible nitric oxide synthase (iNOS) activity, reducing NO production and thereby significantly alleviating edema, hemorrhage, necrosis, and inflammatory responses in cases of acute hemorrhagic and necrotizing pancreatitis [16].

After the SAP model was induced, relative to the M group, the Tet group exhibited lower serum amylase levels, wet/dry weight ratios of pancreatic tissue, pathological scores for pancreatic tissue, and MPO activity levels in pancreatic tissue homogenates. These results indicated that, in the context of SAP, Tet can decrease amylase activity; mitigate pancreatic edema, neutrophil infiltration, and pancreatic injury; reduce the production and release of inflammatory mediators; inhibit the inflammatory response; improve inflammatory immune imbalances; and alleviate pancreatic injury. Thus, through these various effects, Tet can improve the condition of SAP patients. Furthermore, relative to the M group, the Tet group exhibited lower NF-*κ*B transporter activation levels in pancreatic tissue, an increased number of apoptotic pancreatic acinar cells, fewer necrotic cells, and a greater number of surviving cells. These findings suggested that Tet can inhibit pancreatic NF-*κ*B transporter activation, stabilize the intracellular calcium ion levels of pancreatic acinar cells, increase apoptosis among pancreatic cells, reduce necrosis among pancreatic cells, and increase cell survival. Thus, Tet could potentially intervene to ameliorate SAP-related organ damage by affecting multiple targets.
